# Impact of depression on TAVR wait time and post-TAVR outcomes: a population-based study

**DOI:** 10.1093/ehjopen/oeag010

**Published:** 2026-01-27

**Authors:** Zahi Abu Ghosh, Feng Qiu, Ragavie Manoragavan, Derrick Y Tam, Dennis T Ko, Harindra C Wijeysundera, Maneesh Sud

**Affiliations:** Schulich Heart Program, Sunnybrook Health Sciences Centre, 2075 Bayview Ave, D-410, Toronto, Ontario M4N 3M5, Canada; Temerty Faculty of Medicine, University of Toronto, Toronto, 2109 Medical Sciences Building, 1 King's College Circle, Ontario M5S 3K3, Canada; ICES, 2075 Bayview Ave V Wing, Toronto, Ontario M4N 3M5, Canada; Schulich Heart Program, Sunnybrook Health Sciences Centre, 2075 Bayview Ave, D-410, Toronto, Ontario M4N 3M5, Canada; Division of Cardiac Surgery, Department of Surgery, Sunnybrook Health Sciences Centre, 2075 Bayview Ave, Toronto, Ontario M4N 3M5, Canada; Schulich Heart Program, Sunnybrook Health Sciences Centre, 2075 Bayview Ave, D-410, Toronto, Ontario M4N 3M5, Canada; Temerty Faculty of Medicine, University of Toronto, Toronto, 2109 Medical Sciences Building, 1 King's College Circle, Ontario M5S 3K3, Canada; ICES, 2075 Bayview Ave V Wing, Toronto, Ontario M4N 3M5, Canada; Institute of Health Policy, Management, and Evaluation, University of Toronto, 155 College St, 4th Floor, Toronto, Ontario M5T 3M6, Canada; Schulich Heart Program, Sunnybrook Health Sciences Centre, 2075 Bayview Ave, D-410, Toronto, Ontario M4N 3M5, Canada; Temerty Faculty of Medicine, University of Toronto, Toronto, 2109 Medical Sciences Building, 1 King's College Circle, Ontario M5S 3K3, Canada; ICES, 2075 Bayview Ave V Wing, Toronto, Ontario M4N 3M5, Canada; Institute of Health Policy, Management, and Evaluation, University of Toronto, 155 College St, 4th Floor, Toronto, Ontario M5T 3M6, Canada; Schulich Heart Program, Sunnybrook Health Sciences Centre, 2075 Bayview Ave, D-410, Toronto, Ontario M4N 3M5, Canada; Temerty Faculty of Medicine, University of Toronto, Toronto, 2109 Medical Sciences Building, 1 King's College Circle, Ontario M5S 3K3, Canada

**Keywords:** Outcome, Transcatheter aortic valve replacement, Depression, Wait time

## Abstract

**Aims:**

The rising demand for transcatheter aortic valve replacement (TAVR) has outpaced health system capacity, leading to prolonged wait times and adverse outcomes. The impact of pre-existing depression on outcomes while waiting for and after receiving TAVR is not well described.

**Methods and results:**

This population-based study in Ontario, Canada, included all TAVR referrals between April 2018 and March 2023. Depression was identified from provincial physician billing and hospitalization databases. Wait time was defined as the number of days from the TAVR referral to the first of either a TAVR procedure, death, or off-listing from the waitlist for other reasons. The outcomes of interest included mortality and unplanned hospitalization during the waiting period as well as post-TAVR. Outcomes were ascertained to 31 December 2023. We evaluated the relationship between depression and pre- and post-TAVR outcomes using multivariable cause-specific Cox proportional hazard models. The study cohort consisted of 12 789 patients referred for TAVR, of which 3956 (31%) had depression. The median wait time was similar in patients with and without depression (75 vs. 72 days). After adjustment, depression was not associated with differences in the rate of mortality or hospitalization during the waiting period. Similarly, depression was not associated with mortality 1-year post-TAVR. However, depression was associated with a higher rate of unplanned hospitalization post-TAVR (hazard ratio 1.10, 95% confidence interval 1.03–1.17).

**Conclusion:**

Nearly 1 in 3 patients referred for TAVR have pre-existing depression. While depression does not impact wait time-related outcomes, it is associated with unplanned rehospitalization after TAVR.

## Graphical Abstract

**Graphical Abstract:**
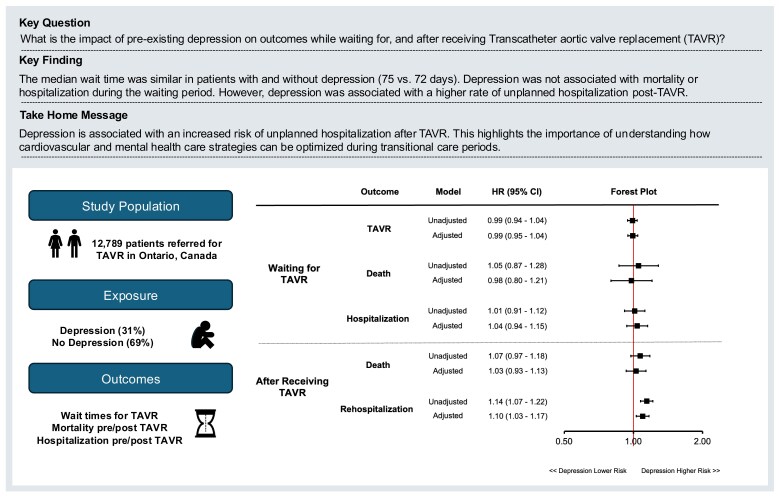

## Introduction

Over the last decade, transcatheter aortic valve replacement (TAVR) has evolved into the first-line treatment for patients with severe symptomatic aortic valve stenosis.^1,2^ This is based on seminal randomized trials showing TAVR is non-inferior to, and in some cases superior to surgical aortic valve replacement.^3–5^ This has translated into an exponential growth in the demand for TAVR, which has overwhelmed capacity in some jurisdictions, translating into prolonged wait times.^6–8^ Prolonged wait times are problematic because they are costly to health systems.^9^ They are also associated with increased mortality as well as hospitalization before^7,10^ and after TAVR.^6,11,12^

Depression is common among patients with cardiovascular disease and is associated with poor outcomes.^13,14^ Two recent studies have suggested that depression is prevalent among patients undergoing TAVR with nearly 1 in 3 patients reporting depressive symptoms.^15,16^ In both studies, depression was associated with increased mortality after TAVR; however, the impact of depression on outcomes while waiting for TAVR has not yet been studied. It is conceivable that pre-existing depression may identify a potentially vulnerable, yet treatable, group of patients undergoing TAVR who may be susceptible to longer wait times and poorer outcomes.

Accordingly, to address this gap in knowledge, we used a population-based cohort of patients with severe aortic stenosis to examine if there exist differences in the wait times for TAVR in those with and without pre-existing depression. We then examined the impact of depression on pre- and post-TAVR outcomes.

## Methods

### Study setting, design, and data sources

Ontario is Canada’s largest province, with a population of 16.1 million.^17^ In Ontario, all residents have universal health care coverage through a third-party payer, the Ontario Ministry of Health. TAVR was first introduced in Ontario in 2007 and began receiving public funding in 2012. Today, TAVR is approved for patients with aortic stenosis across a wide spectrum of risk profiles and is available at all 11 hospitals in Ontario that provide cardiac surgery.

We conducted a population-based, retrospective cohort study of patients referred for TAVR in Ontario between 1 April 2018, and 31 March 2023, using clinical and administrative data sets housed at ICES (previously the Institute for Clinical and Evaluative Sciences). ICES is an independent, not-for-profit research institute. As a prescribed entity under Ontario’s Personal Health Information Protection Act, ICES enables researchers to link clinical registries with deidentified population-based data to conduct approved research studies under regulated privacy and security policies and procedures. The use of ICES data in this project was authorized under section 45 of Ontario’s Personal Health Information Protection Act, which waives the need for review by a research ethics board and individual patient consent.

The primary data source for this study was the CorHealth Ontario TAVR Registry, which collects demographic, comorbidity, and procedural data of all TAVR procedures in the province.^18^ The CorHealth registry was linked to population-level databases that have complete capture for all Ontario residents with a valid health insurance plan number. These databases included: (i) the Canadian Institute for Health Information Discharge Abstract Database (CIHI-DAD) for additional baseline comorbidities as well as outcome hospitalizations, (ii) the Ontario Health Insurance Plan (OHIP) database for physician claims, (iii) the National Ambulatory Care Reporting System (NACRS) for emergency department visits, (iv) the Registered Person’s Database for vital statistics data including the date of death, (vi) the Ontario Mental Health Reporting System (OMHRS) for past admissions for depressive disorders, and (vii) the Ontario Drug Benefit Database which provides prescription medication use among all Ontario residents over 65 years of age. All datasets were linked using unique encoded identifiers and analysed at ICES. We adhered to the Strengthening the Reporting of Observational Studies in Epidemiology statement for reporting of observational studies.^19^

### Study population

All Ontario residents aged 65 years or older, with valid health coverage, who were referred for TAVR from 1 April 2018, to 31 March 2023, were included in this study. The index date for linkage and subsequent analysis was set as the date documented in the CorHealth registry when the referral for TAVR was processed. Referrals are processed within 24 h of receipt of a standardized referral form for TAVR by regional cardiac care coordinators at each of the 11 cardiac centres in Ontario. If a patient had multiple TAVR procedures during the study period, only the first procedure was included.

### Depression and other comorbidities

Depression was defined based on a previously reported algorithm using diagnostic codes for hospitalization/emergency department visits, physician claims (OHIP), or diagnostic codes from the Ontario Mental Health Reporting System.^14^ The presence of depression was ascertained in the 5 years preceding the TAVR referral date using: (i) International Classification of Diseases, 10th Revision (ICD-10) codes F32, F33.1-4, F33.8, F33.9, F34.1, F34.8, F34.9, F38.0, F38.1, F38.8, F39, F41.2 from the CIHI-DAD and NACRS; (ii) physician claims for 300 (reactive depression) and 311 (depression) from the OHIP database; (iii) Diagnostic and Statistical Manual of Mental Disorders, 4th Edition (DSM-4) for mood disorders up to 2016 and DSM-5 for depressive disorders from 2017 onwards and ICD, 9th Revision codes for major depressive disorder, classified as either a single (296.20–296.26) or recurrent episode (296.30–296.36) up to 2018 and ICD-10 codes F32, F33.1-4, F33.8, F33.9, F34.1, F34.8, F34.9, F38.0, F38.1, F38.8, F39, F41.2 from 2019 onwards, as diagnosed by a psychiatrist or attending physician during inpatient admission to a mental health bed using the OMHRS. Validated ICES-derived databases were utilized to identify diabetes, heart failure, hypertension, dementia, and chronic obstructive pulmonary disease.^18^ Medical frailty was defined using the hospital frailty risk score.^20^ Additional baseline comorbidities, including surgical risk defined by the Society of Thoracic Surgeons’ Operative Risk Score, were extracted from the CorHealth registry. Prescriptions for anti-depressants (selective serotonin reuptake inhibitors, serotonin and norepinephrine reuptake inhibitors, norepinephrine reuptake inhibitors, tricyclic anti-depressants, norepinephrine and dopamine reuptake inhibitors, monoamine oxidase inhibitors, noradrenergic, and specific serotonergic antidepressants) in the 1 year prior to referral were assessed among patients 66 years of age and older.^14^

### Outcomes

The primary outcome of our study was the TAVR wait time for patients with and without depression, defined as the time (measured in days) from the TAVR referral to the first of either a TAVR procedure, death, or off-listing from the waitlist for other reasons. The clinical outcomes were all-cause mortality and unplanned all-cause hospitalization. The latter was defined as an emergent or urgent admission to an acute care facility as documented in the CIHI-DAD. Both outcomes were separately assessed during the waiting period, as well as post-TAVR. We also considered cause-specific hospitalizations divided into cardiovascular (using ICD-10 Codes I00-I99) and non-cardiovascular hospitalizations. Post-TAVR outcomes were ascertained from the time of the procedure up to 31 December 2023. Patients who did not experience an outcome were censored on this date.

### Statistical analysis

Continuous baseline characteristics were described with a mean with standard deviation for normally distributed variables and median with interquartile range for variables that were skewed. We developed a Cox proportional-hazards model to assess pre- and post-TAVR mortality for patients with depression compared to patients without depression. A cause-specific hazards model was created for unplanned hospitalization, where mortality prior to the unplanned hospitalization served as a competing risk. All multivariable models were adjusted for demographic factors (age, sex, rural status, and neighbourhood income), medical comorbidities (heart failure, ischaemic heart disease, arrhythmia, peripheral vascular disease, cerebrovascular disease, diabetes, hypertension, dyslipidaemia, chronic obstructive pulmonary disease, dementia, cancer, liver disease, interstitial lung disease, renal disease, and dialysis), Charlson score, frailty score, and prior cardiac procedures (coronary artery bypass surgery, percutaneous coronary intervention, and valve surgery). Post-TAVR models were also adjusted for pre-TAVR wait-time as a continuous linear predictor. Results were reported as hazard ratios (HR) with 95% confidence intervals (CI). We also performed a series of additional and sensitivity analysis. First, to assess if the association between depression was modified by sex, we introduced interaction terms between sex and baseline depression in our fully adjusted models. Second, we stratified rehospitalizations into cardiovascular and non-cardiovascular causes to understand the consistency of the association. Finally, among individuals with baseline medication use, we determine the impact of treatment on outcomes. We classified depression as treated with anti-depressants vs. treated without anti-depressants based on the use of at least 1 anti-depressant in the previous year and compared outcomes to patients without depression. Apart from the Society of Thoracic Surgeons’ Operative Risk Score, which contained missing data and was not included in the regression models, all analyses were conducted on a complete case basis. Data analysis was performed using SAS version 9.4 (SAS Institute, Cary, NC), with statistical significance defined as a 2-sided *P*-value of <0.05.

## Results

### Cohort

**Figure 1:**
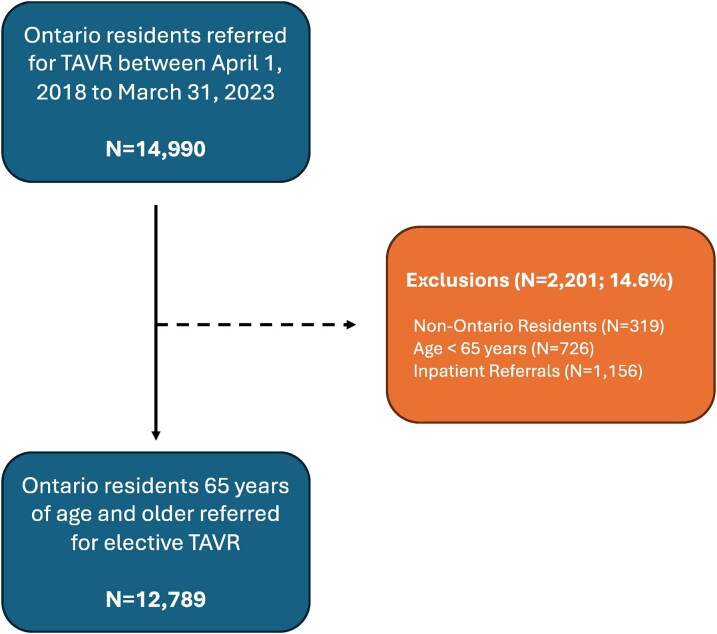
Cohort creation. Flowchart depicts the creation of the study cohort. TAVR, transcatheter aortic valve replacement.

We identified 14 990 TAVR referrals in Ontario from 1 April 2018 to 31 March 2023. After excluding non-Ontario residents (*n* = 319), patients under 65 years of age (*n* = 726), and inpatients (*n* = 1156), there were 12 789 patients remaining for analysis (*Figure 1*). During the follow-up period, 7996 patients (62.5%) received TAVR, 75 (0.6%) remained on the waitlist TAVR, 455 (3.5%) died, and 4263 (33.4%) were off-listed.

### Patient characteristics

Baseline characteristics of the 12 789 patients referred for TAVR are presented in *Table 1*. The mean age of the cohort was 81 years, with 43.7% female and 12.7% living in rural areas. The most common comorbidities were hypertension, dyslipidaemia, heart failure, and diabetes. There were 3956 (30.9%) patients in the cohort with pre-existing depression. A higher percentage of females had depression (51.8% vs. 40.1%), while comorbidities such as dementia (12.0% vs. 5.4%), HF (49.6% vs. 46.0%), chronic obstructive pulmonary disease (18.8% vs. 14.6%), and frailty (19.9% vs. 13.6%) were also more prevalent among patients with depression compared to those without depression. Furthermore, more patients with depression were on anti-depressant medications (42.7% vs. 14.6%).

**Table 1:** Baseline characteristics

Baseline characteristics	Total	No depression	Depression	*P*-value
	*N* = 12 789	*N* = 8833	*N* = 3956	
**Demographics, *n* (%)**				
Age, years (mean ± standard deviation)	81.04 ± 7.05	81.11 ± 7.03	80.88 ± 7.08	0.094
Female	5590 (43.7%)	3539 (40.1%)	2051 (51.8%)	<0.001
Rural	1630 (12.7%)	1206 (13.7%)	424 (10.7%)	<0.001
**Neighbourhood-level sociodemographics, *n* (%)**				
Income quintile				0.009
1st	2665 (20.8%)	1769 (20.0%)	896 (22.6%)	
2nd	2722 (21.3%)	1900 (21.5%)	822 (20.8%)	
3rd	2507 (19.6%)	1784 (20.2%)	723 (18.3%)	
4th	2360 (18.5%)	1641 (18.6%)	719 (18.2%)	
5th	2513 (19.6%)	1724 (19.5%)	789 (19.9%)	
**Comorbidities, *n* (%)**				
Charlson score, mean ± standard deviation	1.13 ± 1.64	1.10 ± 1.62	1.18 ± 1.69	0.015
STS Risk Score, mean ± standard deviation^a^	4.05 ± 3.32	4.05 ± 3.43	4.07 ± 3.08	0.866
Dyslipidaemia	7268 (56.8%)	4899 (55.5%)	2369 (59.9%)	<0.001
Dementia	949 (7.4%)	473 (5.4%)	476 (12.0%)	<0.001
Diabetes mellitus	5487 (42.9%)	3820 (43.2%)	1667 (42.1%)	0.242
Hypertension	11 509 (90.0%)	7918 (89.6%)	3591 (90.8%)	0.049
Heart failure	6031 (47.2%)	4067 (46.0%)	1964 (49.6%)	<0.001
Chronic obstructive pulmonary disease	2030 (15.9%)	1287 (14.6%)	743 (18.8%)	<0.001
Malignancy	853 (6.7%)	567 (6.4%)	286 (7.2%)	0.09
Renal disease	594 (4.6%)	389 (4.4%)	205 (5.2%)	0.053
Coronary artery disease	3508 (27.4%)	2456 (27.8%)	1052 (26.6%)	0.156
Cardiac arrhythmia	1766 (13.8%)	1167 (13.2%)	599 (15.1%)	0.003
Cerebrovascular disease	470 (3.7%)	287 (3.2%)	183 (4.6%)	<0.001
Interstitial lung disease	129 (1.0%)	82 (0.9%)	47 (1.2%)	0.174
Peripheral vascular disease	260 (2.0%)	170 (1.9%)	90 (2.3%)	0.194
Previous percutaneous coronary intervention	2139 (16.7%)	1466 (16.6%)	673 (17.0%)	0.561
Previous coronary artery bypass grafting	1361 (10.6%)	989 (11.2%)	372 (9.4%)	0.002
Previous valve surgery	69 (0.5%)	44 (0.5%)	25 (0.6%)	0.34
Frailty	1984 (15.5%)	1198 (13.6%)	786 (19.9%)	<0.001
**Medications, *n* (%)** ^b^				
Anti-depressant	2960 (23.4%)	1280 (14.6%)	1680 (42.7%)	<0.001

STS, Society of Thoracic Surgeons.

^a^Missing in 68.7% of patients.

^b^Among 12 670 patients referred for TAVR over 66 years of age.

### Wait times and off-list reasons

The median wait time from TAVR referral up to the TAVR procedure, death, or off-listing was similar among patients with and without depression (75 days, IQR: 36 to 129 days vs. 72 days, IQR: 35 to 127 days, *P* = 0.348). A similar proportion of patients on the waitlist with and without depression received TAVR (62.3% vs. 62.6%, *P* = 0.68) or were off-listed (33.4% vs. 33.3%, *P* = 0.86). However, when considering the reasons for off-listing, more patients with depression were off-listed because of their own decision to not pursue TAVR (19.9% vs. 16.1%, *P* = 0.003). On the other hand, fewer patients with depression were off-listed because of a medical decision to not pursue TAVR (69.7% vs. 73.5%, *P* = 0.01). A similar proportion of patients with and without depression were off-listed to pursue surgical aortic valve replacement (9.0% vs. 9.3%, *P* = 0.78) or had an unknown reason for off-listing (1.4% vs. 1.2%, *P* = 0.47).

### Pre-TAVR mortality and unplanned hospitalization outcomes

**Figure 2:**
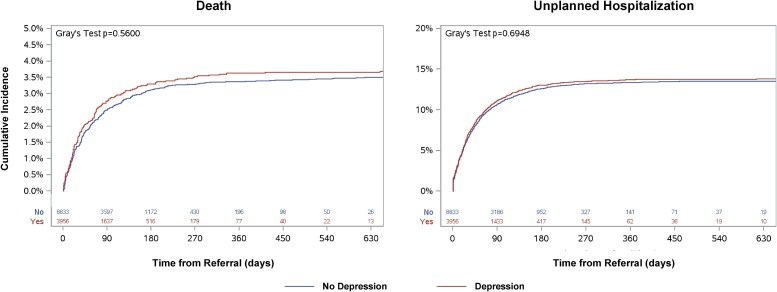
Outcomes while waiting for TAVR. Plots depict the cumulative incidence of death and unplanned hospitalization while waiting for TAVR in patients with (red) and without (blue) depression. Gray’s test indicates equality between two incidence curves. TAVR, transcatheter aortic valve replacement.

There were 3.7% of patients with depression and 3.5% of patients without depression who died while waiting for TAVR. The cumulative incidence of death while waiting for TAVR is displayed in *Figure 2* and did not differ among patients with and without depression (*P* = 0.56). After multivariable adjustment, depression was not associated with mortality while waiting for TAVR (HR: 0.98, 95% CI: 0.80–1.21). Similarly, 13.8% of patients with depression and 13.5% without depression were hospitalized while waiting for TAVR. The cumulative incidence of unplanned hospitalizations did not differ among patients with and without depression (*Figure 2*; *P* = 0.69). Depression was not associated with unplanned hospitalization while waiting for TAVR (HR: 1.04, 95% CI: 0.94–1.15).

### Post-TAVR mortality and unplanned rehospitalization outcomes

**Figure 3:**
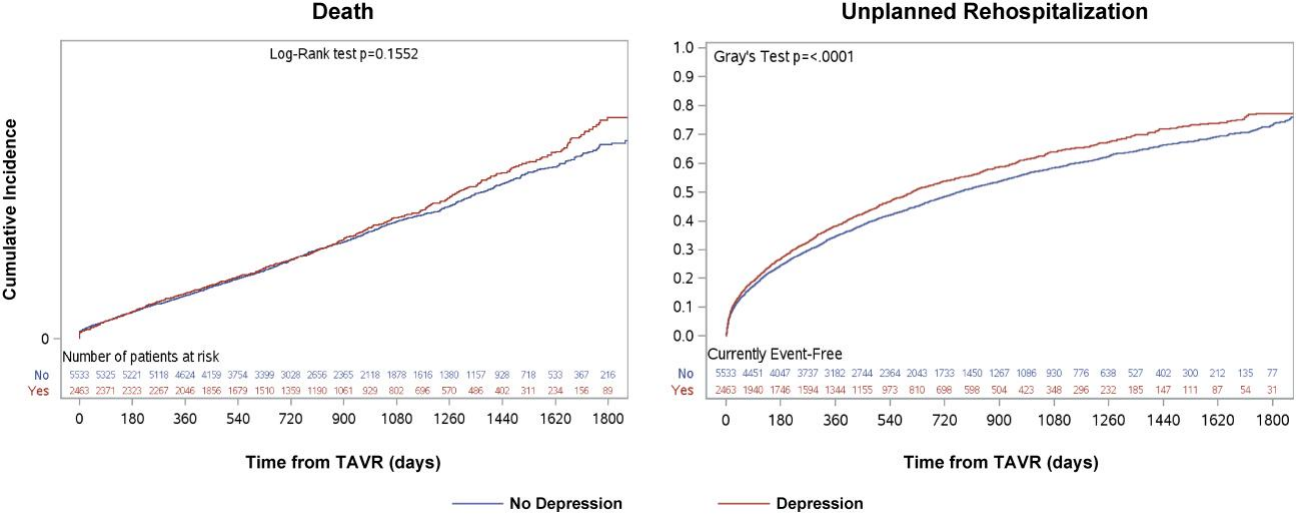
Outcomes after receiving TAVR. Plots depict the cumulative incidence of death and unplanned rehospitalization after TAVR in patients with (red) and without (blue) depression. Gray’s test indicates equality between two incidence curves. TAVR, transcatheter aortic valve replacement.

**Figure 4:**
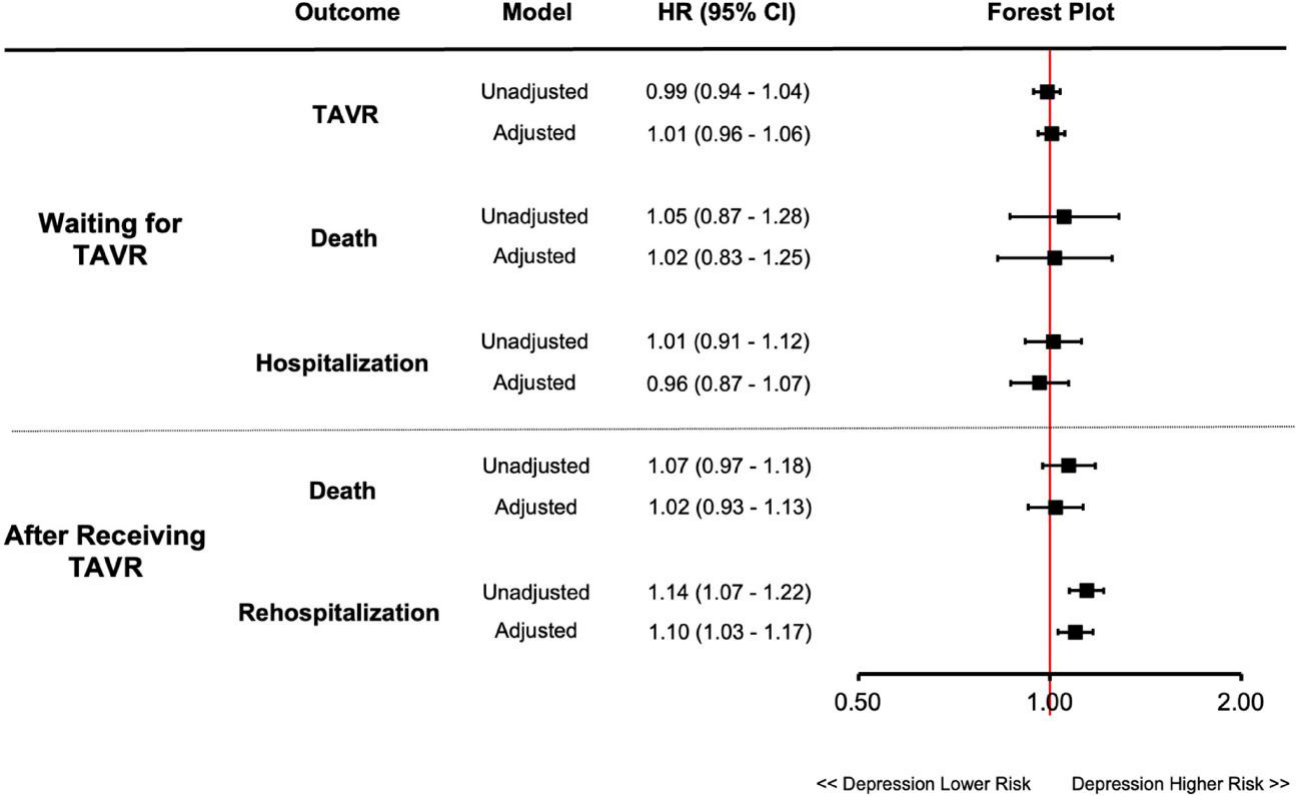
Adjusted association between depression and outcomes. Forest plot depicts the unadjusted and multivariable adjusted association between depression and each outcome. HR, hazard ratio; TAVR, transcatheter aortic valve replacement.

There were 7996 patients who underwent TAVR. During the TAVR hospitalization, few patients required intensive care admission (5.9%), the mean length of stay was 3.5 days (standard deviation 6.2 days), and no differences in these two factors existed based on pre-existing depression status. After the index TAVR procedure, fewer patients with depression compared to those without depression were discharged home without support services (79.7% vs. 82.2%, *P* = 0.01), while more patients with depression were discharged to an alternative inpatient care facility (3.9% vs. 2.9%, *P* = 0.03). Similar proportions of patients with and without depression were discharged home with support services (12.5% vs. 11.2%, *P* = 0.89), were discharged to a group or supportive living environment (1.3% vs. 0.9%, *P* = 0.07), had missing data for dispositions (0.8% vs. 0.9%, *P* = 0.55), or had other dispositions (e.g. left against medical advice, residential care, 0.5% vs. 0.3%, *P* = 0.18). Patients who received TAVR were followed for a median of 789 days (IQR: 454–1249 days). Among the 2463 patients with depression who received TAVR, 9.6% died within the first year. Similarly, among 5533 patients without depression who received TAVR, 9.2% died within the first year. The cumulative incidence curves for death after TAVR over the follow-up period are displayed in *Figure 3* and depression was not associated with post-TAVR mortality after multivariable adjustment (HR: 1.02, 95% CI: 0.93–1.13). On the other hand, the cumulative incidence of unplanned rehospitalizations after TAVR was significantly higher in patients with depression compared to those without depression (*Figure 3*). In the first year, 38.2% and 34.8% of patients with and without depression, respectively, were rehospitalized. Furthermore, after adjustment, depression was associated with a significantly higher rate of post-TAVR unplanned rehospitalization (HR: 1.10, 95% CI: 1.03–1.17; *Figure 4*).

### Additional and sensitivity analyses

First, we found the association between depression status and all outcomes was not modified by sex pre- or post-TAVR (*P*-value for interaction >0.05). Second, the association between depression and cause-specific rehospitalization was determined in patients who underwent TAVR. In the first year, 22.7% and 20.4% of patients with and without depression were rehospitalized for non-cardiovascular causes. On the other hand, 15.5% and 14.4% of patients with and without depression were rehospitalized for cardiovascular causes. The leading causes for readmission at 1 year are presented in Supplementary material online, *Table S1*. Heart failure was the most common cause for cardiovascular rehospitalization and overall, while non-cardiovascular causes were more heterogenous including disorders of the urinary system, musculoskeletal system, delirium as well as unspecified procedural-related complications. Depression was associated with a significantly higher rate of non-cardiovascular rehospitalization (HR: 1.13, 95% CI: 1.05–1.21) and non-significantly higher rate cardiovascular rehospitalization (HR: 1.05, 95% CI: 0.95–1.16). Finally, we classified patients with depression based on the use of at least one anti-depressant agent in the prior year. Compared to patients without depression, neither depression treated with an anti-depressant nor without an anti-depressant was associated with death or hospitalization while waiting for TAVR. Post-TAVR there also existed no association between depression treated with or without anti-depressants and death. However, compared to patients without depression, the association with all-cause rehospitalizations was significant for patients with depression treated with anti-depressants (HR: 1.22, 95% CI: 1.02–1.28) but not those treated without anti-depressants (HR: 1.07, 95% CI: 0.98–1.16).

## Discussion

We used a comprehensive population-based sample to assess the impact of depression on wait times for TAVR, as well as outcomes during and after the waiting period. We found that pre-existing depression was common, and present in nearly 1 in 3 patients referred for TAVR. Depression was not associated with longer TAVR wait times. Interestingly, while a similar proportion of patients with and without depression were off-listed while waiting, more patients with depression were off-listed based on their own decision, while fewer were off-listed based on a decision from their physician not to pursue TAVR. Furthermore, depression was not associated with mortality nor hospitalizations while waiting for TAVR. Yet, depression was associated with a 10% higher risk of unplanned rehospitalizations after TAVR, which may be related more so to non-cardiovascular rather than cardiovascular causes. Our findings identify a large and vulnerable group of patients referred for TAVR, in whom strategies are needed to improve post-procedural outcomes.

Depression is a common mental health disorder affecting up to 30% of the elderly population.^21,22^ In our cohort, pre-existing depression was also common among patients referred for TAVR. We found 31% of patients had depression. While this is comparable with two prior studies that reported depressive symptoms in 20% and 32% of patients undergoing TAVR, several strengths of our study should be noted.^15,16^ First, prior studies used screening questionnaires for depressive symptoms, rather than clinically relevant episodes of depression requiring hospitalization or healthcare utilization. Second, these studies were based on registry data rather than a population-based sample, which is less prone to selection bias. Third, with the rapidly evolving landscape in TAVR, these studies are now, less reflective of contemporary populations referred for TAVR, which tend to include more low-risk individuals.

To our knowledge, this is the first study to evaluate the outcomes of patients with depression waiting for TAVR. Contrary to our initial hypothesis, depression was not associated with longer wait times for TAVR. Moreover, we found no significant differences in outcomes between patients with and without depression awaiting TAVR. Interesting, we noted up to 33% of patients were off-listed, and depression is more common in patients who chose to off-list rather than pursue TAVR. Depression is known to impair self-care, reduce adherence to medical recommendations, which may lead to increased long-term healthcare utilization. Our findings may reflect the reduced motivation, lower health engagement, and poor adherence to medical care in this vulnerable group. This highlights the importance of understanding how the use of TAVR among patients who are medically eligible may be optimized using a patient-centric approach.

Despite similar wait times and pre-TAVR outcomes, depression was associated with a significantly higher risk of unplanned rehospitalization post-TAVR. While prior studies of depression in the TAVR population did not report rehospitalization outcomes in their analysis, these findings remain consistent with the higher risk observed in depressed patients with coronary artery disease and heart failure.^13,14,23^ Furthermore, we noted that the risk of non-cardiovascular, rather than cardiovascular rehospitalization was higher after TAVR in patients with depression relative to those without depression. Explanations for the discordance in hospitalization risk for the pre- and post-TAVR periods are not entirely clear. It is conceivable that this may be related to the intensity of health care contact as well as deficiencies in transitional care strategies. Once referred for TAVR, patients with severe aortic stenosis are often co-managed at specialized TAVR clinics with multiple contacts with healthcare professionals (e.g. outpatient visits, diagnostic testing, invasive angiography procedures), which may mitigate unplanned readmission risk while waiting for TAVR in patients with depression. However, once the TAVR procedure is completed and patients are discharged from hospital, transitional care challenges, which may not represent deficiencies in post-TAVR care pathways *per se*, may have disproportionately affected patients with depression, contributing to the observed higher percentage of unplanned hospitalization, despite the similar wait times, and outcomes pre-TAVR. Hence, understanding unmet health-related social needs for individuals with depression that contribute to poor integration back into the community setting after discharge (e.g. lack of psychosocial support, access to community health workers, health-related education, and counselling) may provide opportunities to build targeted transitional programmes to reduce rehospitalization burden.^24^

On the other hand, we found that depression was not associated with increased mortality following TAVR, which is in contrast to findings from recent studies.^15,16^ We believe this may be explained by methodological differences in our analysis. We included an extensive list of additional confounders such as, but not limited to, frailty, the Charlson comorbidity index, and chronic obstructive pulmonary disease. These have been shown in previous studies to influence post-TAVR outcomes^25–27^ and are more prevalent among patients with depression. Hence, adjusting for them may have attenuated the observed association between depression and mortality after TAVR.

### Limitation

There are several potential limitations of this study that merit consideration. First, the identification of patients with depression was not based on formal psychiatric assessments. We did, however, use diagnostic codes representing individuals with episodes of depression requiring admission and inpatient treatment. Second, our analysis was conducted in Ontario, Canada, in which patients receive care under a universal health plan. Hence our findings, may not be generalizable to other healthcare systems. Third, clinical overlap between depression and cognitive impairment may exist in older individuals. While we adjusted for both using administrative definitions, formal cognitive assessments were not available in our cohort. Fourth, our secondary analysis of cause-specific hospitalizations post-TAVR may be subject to misclassification, particularly for procedure-related complications. Finally, despite extensive adjustment using both prospectively clinical parameters from CorHealth registry and supplementation with comorbidities using validated administrative algorithms, we cannot rule out the presence of residual confounding.

## Conclusion

In conclusion, our study found that nearly 1 in 3 patients referred for TAVR had pre-existing depression. While depression was not associated with an increased risk of unplanned hospitalization or mortality during the waiting period for TAVR, we found it was associated with an increased risk of unplanned rehospitalization after TAVR. Our results also suggest, this may be attributable to an increased risk of non-cardiovascular, rather than cardiovascular rehospitalizations. Our findings highlight the importance of understanding how cardiovascular and particularly, broader mental health care strategies can be optimized during transitional care periods post-discharge from the TAVR procedure to improve outcomes in this vulnerable population.

## Supplementary Material

oeag010_Supplementary_Data

## Data Availability

The dataset from this study is held securely in coded form at ICES. While legal data sharing agreements between ICES and data providers (e.g. healthcare organizations and government) prohibit ICES from making the dataset publicly available, access may be granted to those who meet pre-specified criteria for confidential access.
