# Supplementary material for: Impact of depression on TAVR wait time and post-TAVR outcomes: a population-based study
Source: Eur Heart J Open. 2026 Jan 27;6(1):oeag010. doi: 10.1093/ehjopen/oeag010 (PMC12930845; doi:10.1093/ehjopen/oeag010)
Supplement: oeag010_Supplementary_Data [file oeag010_supplementary_data.docx]

**Impact of Depression on TAVR Wait Time and Post-TAVR Outcomes: A Population-Based Study**

**Supplementary Material**

Zahi Abu Ghosh, MD et al.,

**Supplementary Table 1. Leading Diagnosis for Rehospitalization after TAVR**

| **Depression** | | | **No Depression** | | |
| --- | --- | --- | --- | --- | --- |
| **ICD-10 CA Code** | **Diagnosis Description** | **Proportion (%)** | **ICD-10 CA Code** | **Diagnosis Description** | **Proportion (%)** |
| I50 | Heart failure | 9.2% | I50 | Heart failure | 9.2% |
| I44 | Atrioventricular and left bundle-branch block | 4.6% | I44 | Atrioventricular and left bundle-branch block | 5.4% |
| I63 | Cerebral infarction | 2.8% | I63 | Cerebral infarction | 3.3% |
| N39 | Other disorders of urinary system | 2.3% | I21 | Acute myocardial infarction | 2.3% |
| T81 | Complications of procedures, not elsewhere classified | 2.3% | T82 | Complications of cardiac and vascular prosthetic devices, implants and grafts | 2.1% |
| F05 | Delirium, not induced by alcohol and other psychoactive substances | 2.2% | M17 | Gonarthrosis [arthrosis of knee] | 2.1% |
| I48 | Atrial fibrillation and flutter | 2.0% | S72 | Fracture of femur | 2.1% |

Diagnoses in red indicate cardiovascular causes
